# Healthy Mobile Work: The Relationship of a Participative Work Agreement and Workplace Health Management-Qualitative Results of a Longitudinal Study

**DOI:** 10.3390/ijerph19127526

**Published:** 2022-06-20

**Authors:** Marlies Jöllenbeck, Olivia Maloku, Ines Berling, Tjorven Stamer, Elke Ochsmann

**Affiliations:** 1 Institute of Occupational Health, Prevention and Workplace Health Management, University of Luebeck, 23562 Luebeck, Germany; ines.berling@student.uni-luebeck.de (I.B.); tjorven.stamer@uksh.de (T.S.); elke.ochsmann@uksh.de (E.O.); 2 Workplace Health Management, Statutory Accident Insurance (Unfallkasse Nord), 24113 Kiel, Germany; olivia.maloku@uk-nord.de

**Keywords:** mobile work, work agreement, workplace health management, health-oriented work design

## Abstract

Mobile work is becoming increasingly common, and it has been, consequently, associated with new health-related hazards and resources. Our study examined health-related stresses, strains and resources of mobile work in a medium-sized company. The study aimed to generate implications for a work agreement and for workplace health management (WHM). For this, a multi-method longitudinal study (2019–20) was conducted, with 29 focus group and 6 individual interviews (absolute number of all participants *N* = 187). It was designed as a qualitative content analysis and theoretically based on the job demands-resources model (JD-R). Positive effects (e.g., increased work–life balance, higher concentration), as well as negative consequences (e.g., alienation in the team, communication effort), can be found. Numerous fields of action for both the work agreement and WHM could be identified. For example, the work agreement regulates the equipment for working from home with support from WHM in order to ensure occupational health-oriented selection and handling, or by fixing core working hours through the work agreement and supporting competence building for leaders in order to enable flexible work commitments for employees. Self-organised work at home can be supported both by rules in the service agreement and by building up self-management skills through the WHM’s offers. The findings illustrate that a work agreement can make a relevant contribution to a healthy design of mobile work by systematically linking it with WHM. The synergies between work agreement, employee health and WHM become clear.

## 1. Introduction

### 1.1. Background, Definition and Effects of Mobile Work

New technologies and digitalization processes are fundamentally transforming the world of work: work content, work organization, the work environment and even the place of work [1] are changing, increasing flexibility with global networking across companies and national borders through digitalisation, summarised under the buzzword Industry 4.0, and promoting location- and time-independent working in new, mobile forms of work [2,3,4]. In particular, activities in the service sector and knowledge-intensive work are increasingly replacing physical workplaces [5]. The COVID-19 pandemic has further accelerated this development [6].

Means of information gathering and new communication channels offer a variety of new ways for shaping work [7]. Technological developments drive change in the world of work and lead to organisational changes taking place at ever shorter intervals [6]. This leads to new or changed hazards and resources in the workplace [7,8].

The term mobile work has a variety of definitions. One definition is that employees spend 10 h or 20% of their week working away from the workplace or company office, using new information technologies [9]. A distinction can be made between work-associated and work-related forms of mobility [9]. In the case of work from home, a distinction can be made between teleworking and kinds of home offices. A characteristic of teleworking is that employees have a fixed workplace at home, which is often set up with the support of the employer. The teleworking model is subject to the health and safety regulations of the workplace ordinance [3,9]. For the less regulated and formalised forms, the terms home office or working from home (WFH) are established [3]. This study uses the term “mobile work” to refer to all the mobile forms of work just mentioned. Against the backdrop of the COVID-19 pandemic and the associated lockdowns, however, there have been findings on WFH in particular.

In the literature, mobile work is mostly discussed from a microergonomic perspective, focusing in particular on the experience of strain and stress [1,7,10]. Health effects of mobile forms of work include, for example, increased satisfaction, reduced experience of stress in the professional role [11] and a reduction in general physical and psychological stress [12,13], but an increase in depressive symptoms and exhaustion, musculoskeletal complaints and coronary heart disease is also observable [14,15,16]. The health effects of new forms of work are moderated by organizational variables, such as work intensity, leadership behavior and work equipment for WFH [14,15]. 

However, a deficit can be identified at the macro-ergonomic level in terms of, in particular, occupational health and safety (OHS) [3,4]. Mobile forms of work with physical separation are changing access to workers. This requires new strategies from institutional actors such as leaders and OHS experts. In this context, a lack of appropriate tools for the organisation of flexible working arrangements, as well as for their risk assessment, can be noted [4]. Furthermore, it is considered difficult to enforce rules and regulations at the institutional level [3].

The dynamic developments in a fast-changing world of work make healthy work design challenging for companies and internal actors. Systematic procedures and control instruments are needed for this. For example, questions on the management of safety and health in flexible working environments can only, so far, be answered inadequately [3].

### 1.2. Work Agreement and Workplace Health Management

#### 1.2.1. Work Agreement

Work agreements are a well-established, but not necessarily health-related procedure between employers and employee representatives in Germany. They regulate the legal relationship between the employer and the employees regarding internal matters not covered by general regulations [3,7]; e.g., mobile work. Work agreements are typically used to establish framework conditions on specific work contexts such as organizational arrangements for mobile work (core working hours, accessibility), the entitlement to work equipment for telework, work content, the place of work, the accessibility of the employee and the organisation of working from home (WFH) [17]. Work agreements are agreed on the basis of German labour-related laws [18]. The work agreement can therefore be described as a classic, administrative instrument of management in companies. 

A review from Germany, in which 31 work agreements were included in the analysis, was able to show a broad spectrum of contents and agreements [17]. Aspects of OHS are also addressed in various ways [17]. In addition, there are work agreements that regulate occupational health management as an independent field of action [19]. However, important aspects, such as leadership and corporate culture, are currently hardly reflected in work agreements [3]. In a meta-analysis, no negative effects were found between mobile work and social relationships with managers [11]. However, leadership is undoubtedly a relevant adjusting screw both for work design and for the health and safety of employees, as shown, for example, in the meta-analysis by Montano et al. (2016) [20] and other studies [3,21,22]. The proportion of mobile workers who work autonomously and away from their company’s main sites for at least part of their working time is growing and is characterised by distance in terms of time and space [21,23]. However, many leadership styles and management systems are still focused on direct interaction at the office [21]. A 2018 systematic review of 23 papers shows that both management and leadership are important in ensuring OSH for mobile workers [21].

An evaluation of the implementation of location and time flexible working is increasingly in demand due to the growing relevance of the topic [17,21]. This involves the question of how individual employees work, what helps them or what would still be necessary for a smooth implementation of mobile work [7,10]. These are as yet unanswered questions which, in the view of the authors of this study, are of great importance for the health and occupational safety of employees under changing conditions and point to the close reciprocal relationship between work agreements and WHM. 

#### 1.2.2. Workplace Health Management (WHM)

WHM is also a management approach, with a central focus on the health of employees. WHM is the systematic data-based development, planning and steering and strengthening of company structures and processes with the aim of maintaining and promoting the health of employees [13,24]. In practice, workplace health promotion (WHP) and workplace health management (WHM) are often used synonymously. According to the definition of the European network for Workplace Health Promotion (ENWHP), workplace health promotion includes all joint measures of actors to improve health and well-being at the workplace [25,26]. Workplace health promotion can include both systematic approaches and non-systematic individual measures. In the understanding of this study, a definitional distinction is made between WHM and WHP, since workplace health management is strategically integrated as a management approach into company structures.

Four dimensions are described as central: integration into company policy, planning, support and evaluation of health-related activities in the company [27]. This emphasises that occupational health management should be anchored in all fields of action of a company in order to understand and implement health as a cross-sectional task [26].

#### 1.2.3. The Link between the Work Agreement and WHM

Both approaches—work agreements and WHM—exist together in many companies, although a linkage in terms of health-oriented work design seems to make sense. The benefits, effectiveness and health-economic efficiency of a WHM have been proven by a number of studies [26,28]. However, there is a lack of knowledge on how WHM can be integrated into existing management processes. According to Faller (2018), there is a particular lack of qualitative research on the “how” of integrating WHM into companies, taking into account the specific organizational culture [26]. With this study, we would like to make a contribution to narrowing this research gap.

Whether and in what way a work agreement can contribute to WHM, or, conversely, WHM methods could be helpful for the development of work agreements, has not yet been investigated in detail according to current knowledge. The present study therefore examines how a link between administrative work agreements and WHM can be established in the context of mobile work in order to create healthy working conditions.

### 1.3. Background and Purpose of the Study 

The foregoing aspects provide the basis and focus for the study results presented here.

The purpose of this study is to examine the intersection between a work agreement and WHM.

Specifically, in this paper we would like to shed light on the way in which a work agreement can contribute to WHM or, conversely, WHM methods could be helpful for the development of company agreements in order to strategically anchor health in all fields of action of an organisation. According to the authors’ current knowledge, this has not yet been investigated in detail. 

The starting point and data basis is a longitudinal study in a public administration with almost 200 employees, in which the effects of mobile work were investigated using a multi-method study design. The aim of the company was to generate essential criteria for the design of a work agreement for mobile work by means of a participatory process. In addition, measures for occupational health management related to mobile work were to be determined. The main qualitative results from 29 focus group interviews and 6 individual interviews are presented here.

## 2. Methods

### 2.1. Research Design

#### 2.1.1. Qualitative Research

The qualitative research was part of a mixed-method approach of a longitudinal study. The mixed-method approach is a research approach that has been established for many years, in which qualitative and quantitative research strands are combined in order to achieve the research goal in the best possible way [29,30,31]. This approach is particularly suitable for the exploration of research fields and change processes [32]. While the quantitative survey method is suitable for collecting standardised opinions, attitudes, interests, etc. from larger groups of people, the semi-structured, qualitative interview in combination with the focus group method is suitable for delving deeper into a topic area and revealing experiences, special features, problems or even problem-solving strategies in a specific context [32]. It is of central importance to activate “tacit knowledge” through discussion and to broaden perspectives on a topic [32,33,34].

Qualitative data was collected through focus group interviews and individual interviews at two points in time. At T0 (Nov 2019), the focus groups were composed of a test group to trial mobile working in the company. At T1 (Aug 2020), almost all employees had experienced working from home as part of the COVID-19 pandemic and could be included. 

The focus group interviews in both survey waves were designed to record the experience of stress and strain, as well as experiences, perceptions and coping with mobile work and the change process by means of a participatory procedure over time. The aim was to generate relevant and practical design criteria for a future work agreement and impulses for a health-oriented design. 

The guiding questions of the first survey wave were initially aimed at comprehensively exploring the work situation in relation to mobile work. The aim was to gain a better understanding of the factors influencing mobile work on the behaviour and health of employees. The first round of questioning was intended to generate the identification of relevant influencing factors on the healthy design of mobile work, as well as the design criteria, which were then focussed on and deepened in the course of the second wave of questioning.

#### 2.1.2. Questionnaire Design

The semi-standardized interview guide follows the guidelines of qualitative social research [34,35]. The first qualitative survey (T0) was based on the five fields of action of the guideline of the Joint German Occupational Safety and Health Strategy, described in Kap. 2.2.2. The guideline makes it possible to comprehensively map the work situation and was supplemented by aspects of advantages and disadvantages of mobile work. 

**Figure 1:**
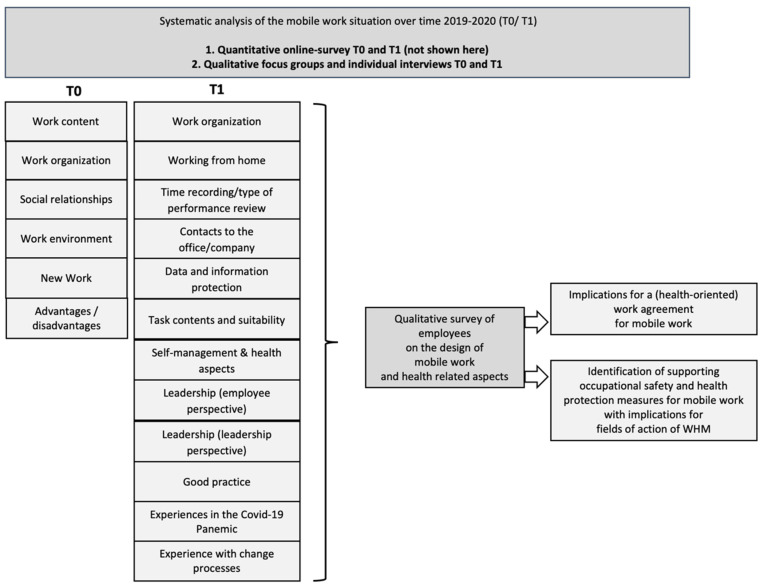
The procedure and representation of the iterative, qualitative research process.

In the sense of a cyclical-iterative research process [36], the focus of the second qualitative survey was on identifying changes between T0 and T1 and on the experience of mobile work. In the interview guide at T1, the dimensions of an exemplary work agreement and questions regarding the COVID-19 pandemic were also included (Figure 1). 

### 2.2. Theoretical Foundations

#### 2.2.1. Job Demands-Resources Model (JD-R)

The Job Demands-Resources model (JD-R) by Demerouti et al. brings together job design models with stress-strain models and combines these research approaches [37,38]. The combined consideration of job demands and motivational factors enables us to understand job demands and resources both independently and in interaction with work-related aspects such as burnout and work engagement [37]. The model includes the embedding of the job crafting model [37,38]. Job crafting is described as the ability of employees to use the creative leeway available to them and to shape work to suit themselves [37]. Positive effects of job crafting are an increase in satisfaction and a sense of meaning. In times of high flexibility, diversity and growing levels of uncertainty and complexity in the workplace, it is becoming increasingly important for employees to be able to shape their work in order to make it fit [39].

From our point of view, this model is particularly suitable for this study because we aimed to identify concrete criteria for a healthy design of mobile work. 

#### 2.2.2. German Guideline of (Mental) Health and Safety 

The German Guideline of Mental Health and Safety is an important instrument of occupational health and safety [40]. It represents the Joint German Occupational Health and Safety Strategy and was established by the Federal Government, states and the accident insurance institutions [40]. The guideline enables a systematic procedure to identify stress in the workplace. Five fields of action are considered: work content, work organisation, social interaction, working environment and new forms of work [40]. This guideline was an important theoretical and methodological basis of the study design.

### 2.3. Study Setting, Participants and Sampling Procedere

#### 2.3.1. The Company

The study was conducted in a medium-sized company in the social security sector. At the time of the survey, 193 people were employed there. About 70% of all employees in the company were employed full-time, about 30% part-time. At 60%, slightly more women than men were employed there at the time of the survey. Almost half of all employees were in the age group > 50 years. The company is represented at several locations. A large number of employees are exclusively engaged in administrative activities at the office. Another group works both in the office and in the field. The work content consists of office and administrative activities, consulting and support of companies, as well as numerous administrative activities. According to the assessment of the management, the work content in all departments is predominantly suitable for mobile work. Only a few areas of activity would not be suitable for mobile work according to the current status, such as the information center and the post section.

In 2019, a test run for mobile work was started in the company for a test group of almost 30 employees. Participation in this pilot phase was voluntary. The company’s initial intention was to gradually establish mobile working as a new form of work for the employees. The study was intended to help the company gain an understanding of the change process, to explore the company’s internal needs for mobile work and to generate starting points for a health-friendly design. During the COVID-19 pandemic, almost all employees were induced to work from home during the lockdowns.

#### 2.3.2. Sampling Procedure and Participant Recruitment

In the first round (T0), the focus was on interviews with people of the pilot-phase and their direct colleagues. The sample for T0 was defined using the following inclusion criteria: Participants should be at least 18-years-old in the first round (T0), the focus was on interviews with persons of the pilot-phase and their direct colleagues within the team. This concerned colleagues in a team or even in an organizational unit.

Due to the expansion of WFH in the context of the COVID-19 pandemic, a company-wide invitation was issued in T1, without exclusion criteria except for age under 18 years. 

The participants were recruited through an internal procedure on the part of the company through information by the managers, as well as by mail. 

Participation in the focus groups was voluntary at all times. The interviews took place during working hours. Participants signed a written consent form and were fully informed in advance in writing, as well as verbally at the beginning of the focus group interviews about the aim and purpose of the study, as well as the anonymity of the procedure.

The focus group interviews were arranged according to teams and organisational units. Middle and senior management levels were brought together in a separate focus group (*n* = 11). The group size was between 4–10 people.

#### 2.3.3. Research Setting and Conduct of the Focus Group Interviews 

All focus group interviews took place outside the company in the conference room of a hotel. The working atmosphere was pleasant and relaxed. Participation in the interviews was working time. 

The participants gave their written consent for the session to be audio recorded directly before it started.

The facilitators were qualified and experienced in conducting focus group interviews and individual interviews. The research team members (M.J., T.S., I.B.) were mostly present in pairs in the sessions for quality assurance reasons.

The advantage of focus group interviews is to enable a multi-layered examination of the topic and a variety of aspects and perspectives. Contexts of interpretation and action can be developed through the statements of the participants [41]. Emotional backgrounds of statements and hidden assumptions are thus revealed. Possible disadvantages are when individual interlocutors dominate the conversation. Various methods were used to prevent this phenomenon, for example by explaining the use and purpose of focus group interviews again [41,42]. A trusting atmosphere was created by acknowledging the contribution of all those present as necessary and important on the part of the moderator. Supporting rules for the joint discussion were introduced, such as letting each other finish and mutual respect for different opinions. Speeches were balanced in time so that all participants were actively involved in the discussion. Methodologically and in terms of content, the workshops were organised along the focal topics (see Table 1). The discussion topics were presented and explained briefly, without giving too much guidance, so as not to create tendencies in the opinions and the discussion.

The interviews followed a semi-standardised procedure and a guideline (see Figure 1 for topics). The answers were visibly documented on a whiteboard for the participants. Point by point, it was agreed with the interviews whether the documentation was correct to confirm the reliability and validity of the data in terms of the qualitative research approach.

### 2.4. Data Analysis and Quality Standards

The focus group interviews were recorded (logged in 3 cases) and professionally transcribed. The qualitative content analysis according to Mayring [42] was carried out with the text analysis software MAX-QDA, version *Analytics Pro 2020*. Coding using coding guidelines was designed as a structuring, deductive analysis process. For quality assurance (inter- and intra-coding reliability, transparency, coverage), this was carried out by two researchers (M.J. and T.S.) [43,44]. In order to ensure the reliability and validity of the coding process, the evaluation process followed a fixed procedure in which the members of the team create and check the codes sequentially and twice. Findings were discussed with all group members, e. g., an occupational physician (E.O.), a WHM-expert (O. M.) and external experts (social insurance experts, members of the companies’ workers council, representative for employees with special needs). New categories were allowed in the sense of inductive exploration of the field in order to maximize the gain of knowledge. Reporting follows the criteria of the COREQ checklist (Consolidated criteria for reporting qualitative research) [44] and standards for reporting qualitative research [45]. 

### 2.5. Transfer of Content between Service Agreement and WHM

As part of the data analysis process, the results T0 and T1 were discussed and classified in an interdisciplinary expert group with regard to their relevance for the company. The expert group consisted of the leader, the WHM representative, OSH experts, employee representatives and the researcher. The classification took place in two steps: 1. The results were discussed with regard to their significance for the work agreement to be developed, 2. WHM measures were determined from the contents in order to support and accompany the process of introducing mobile work. 

### 2.6. Data Protection and Ethical Aspects 

Data protection, as well as ethical and scientific standards, were fully taken into account. The evaluation is completely anonymous. It is not possible to draw conclusions about individual people. Participation in the interviews was voluntary.

A positive vote of the ethics committee of the University of Lübeck is on hand.

## 3. Results

### 3.1. Sample 

A total of N = 187 persons (T0 and T1) participated in 29 focus groups and 6 individual interviews (individual interviews only in T1 necessary because of the lockdown of COVID-19 pandemic) (Table 1). If organisationally possible, teams or groups were grouped by organisational unit. Within the groups, there were people with very different work characteristics, for example in terms of fields of activity, work content, office and field work. At T0, it was possible to differentiate between people with and without experience of mobile work. After the start of the COVID-19 pandemic, all participants had experience with mobile work respectively working from home at T1. About 80% of participants in T0 also participated in T1.

### 3.2. Overview of Categories and Frequencies of Codes

**Figure 2:**
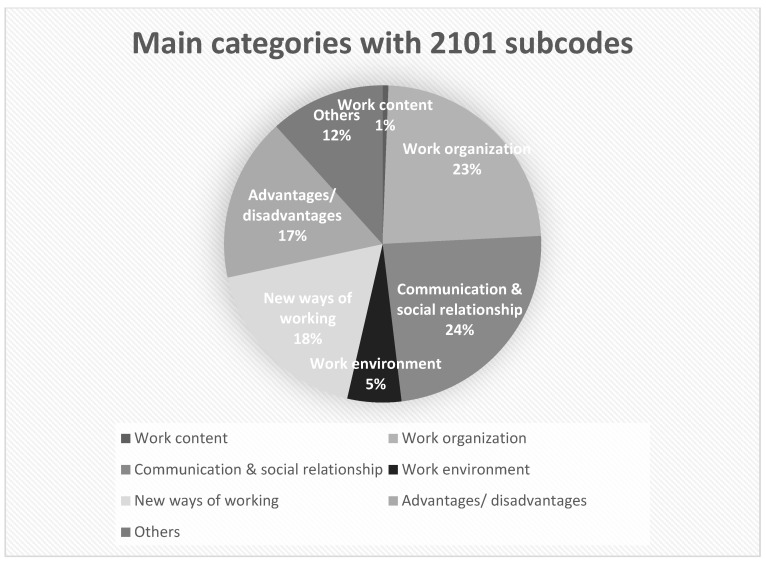
Categorical frequencies of T0.

**Figure 3:**
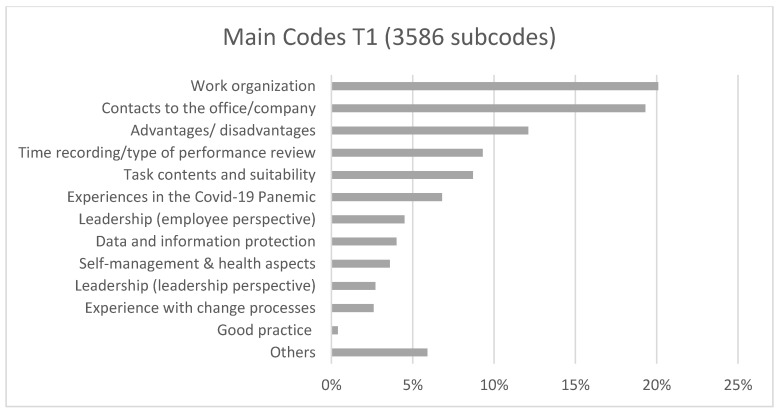
Categorical frequencies of T1.

Within the framework of the content analysis, 16 main categories with 2101 individual codes were generated in T0. Figure 2 lists the 6 most important items; the rest were summarised under “Other”. In T1, 18 main codes with 3586 subcodes were identified. In Figure 3, 13 items are listed, the rest with individual mentions were summarised under “Other”.

The most frequently mentioned aspects at both survey times were work organisation, social interaction, advantages and disadvantages (also health-related) and the influences of the COVID-19 pandemic (T1 only).

The most frequently mentioned categorical main codes are presented in detail below and placed in the context of a work agreement and the WHM. The results listed are supported by so-called anchor examples using original quotes from the interview partners. The translation of the original quotes was checked by a native English speaker by means of back-translation.

### 3.3. Content of Work

With regard to the work content, hardly any changes between mobile work and no mobile work in their own activities were perceived in the context of T0, nor were any expected in the future. However, the recommendation to take a good look at the interface areas was already formulated in the first round, because changes are to be expected with the company-wide expansion of mobile work. The post section was mentioned as an example of an interface. In the second wave of the survey, changed or even strongly changed work content could be observed in the context of the pandemic, for example because customer contact had to be eliminated. However, the employees’ assessment here was that this could not be assessed at the time of the survey due to the distorted conditions caused by COVID-19 pandemic.

### 3.4. Work Organisation

Many of the respondents’ comments could be categorised under the heading of work organisation. With regard to work organisation, the respondents distinguished between the requirements of self-organisation and work organisation on the part of the company. With regard to company work organisation, numerous requirements were mentioned to organise and secure work processes at different work locations. 

For mobile working, complete access to documentation systems is necessary and insufficient digitalisation of the individual organizational units makes work processes more difficult. A clear need was formulated to organise working hours in such a way that they meet both the advantage of flexibility and the need for reliable accessibility. “*There needs to be a balance between structure and flexibility. In my opinion, this is a process and cannot be primarily fixed. We first have to find out together with our leader what a suitable way would be*” (Interview T0). The situation was described as particularly complex for staff and their colleagues when mobile work involves both fieldwork and WFH. In the discussion, it became clear that more effort is needed in coordination within the team and especially with interdisciplinary areas. The direct colleagues of the persons in T0, who were not mobile workers themselves at that time, reported an increasing workload, especially because coordination and the need for clarification were delayed.

*“There is still a lot to clarify because I cannot always leave my own work immediately just because the colleague brings me documents that need to be processed. Of course, that used to be much easier when we sat next to each other”*

(Interview T0)

The following organisational aspects emerged for which the interviewees primarily already expressed the expectation that these would be the subject of the future work agreement.

Effect of work equipment on work processes:

The furnishing and equipment of the workplaces (especially the technical equipment) is closely connected to the organisation of work, for example with regard to scheduling, performing documentation tasks *“between two field service appointments”* (Interview T0) and access to printers and the internet. The desire for equipment that meets needs was expressed in many cases.

*“What is really needed should be there so that the work can be done. But not everything is needed for everyone”*

(Interview T0)

Time recording/performance quotas:

A clear regulation and practicable recording of working time as well as performance monitoring was often desired by the employees themselves in order to create a transparent work situation. The future performance evaluation should be result-oriented based on clearly defined criteria. The concretization of the criteria was associated with expectations of a culture of trust.

*“You can’t cast everything in rules. Without the trust of leaders, the whole construct of mobile work doesn’t work”*

(Interview T0)

The support of managers and the quality of cooperation between employees and managers were named as important points regarding the recording and evaluation of performance.

Sphere of action:

For the scope of the work agreement on the use of mobile work, the need for transparent criteria regarding the suitability of activities and persons was emphasized. Eligibility criteria as well as inclusion and exclusion criteria for mobile work must be clearly regulated and communicated. In principle, however, mobile work should be made available to all employees on a voluntary basis. Linked to this was the wish to have a clear description and definition of mobile or home-based work in order to prevent misunderstandings.

*“So, there should be a compulsion to have a home office again, nor should individuals be excluded. This must be viewed in a differentiated way. It has a lot to do with justice that everyone gets the chance to work flexibly. But of course, people must also realise if their workplace is not suitable for this”*

(Interview T0)

Self-organisation and individual work design competence:

Mobile work goes hand in hand with a significantly increased need for self-organisation (e.g., break management, work motivation). The personal demarcation between “work” and “private” must be actively shaped, for example through rituals (clothing like in the office) and regulated working hours, spatial demarcation of the work area. Health self-care is easier in the home, for example through more flexible movement options.

*“Yes, that is definitely one’s own organization. So (…) that comes out as a side effect, how you deal with yourself, that is, how you deal with your own person*

(Interview T1)

### 3.5. Social Interaction/Contact with the Office

Social interaction and contact with the workplace were relevant at both interview times. 

In general, different positions could be identified. While a significantly larger group had a negative connotation of a reduction in social contact due to mobile work, there were also a few employees who felt no loss of contact at all. These were mainly people who already had a lot of experience with mobile work and primarily had experience in the field.

*“I really miss the contact with my colleagues. It’s just nice and pleasant to talk about topics beyond work and to laugh together sometimes.”*

(Interview T0)

**Figure 4:**
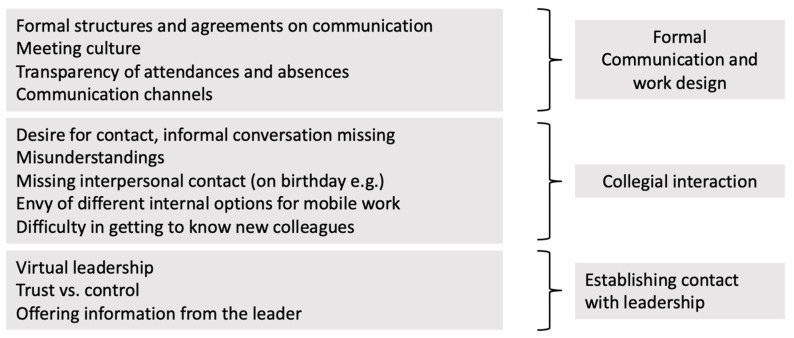
The differentiation of the content of the main code “social interaction”.

Three dimensions of social interaction could be distinguished: the “formal” organisation of work and communication, the collegial, informal interaction and the organisation of contact with leadership (Figure 4). 

With the introduction of mobile work, an *“immense reduction in contact”* (Interview T1) with colleagues was experienced. This was predominantly evaluated negatively, especially in the area of personal, informal communication. Informal or “accidental” communication, which is important for the casual exchange of professional information, would be largely reduced by the introduction of mobile work. The provision or improvement of transparent information and communication structures was desired in order to prevent misunderstandings and conflicts. The interviewees also mentioned the importance of clearly defining responsibilities, roles and tasks, especially at interdisciplinary interfaces. The provision of suitable rooms in the office for regular personal exchange among each other could strengthen exchange, the working atmosphere and identification with the company. It turns out that interdisciplinary cooperation in particular suffers from mobile work—this is less of a problem for team-internal communication. 

Great influence on social interaction is attributed to leadership—especially in virtual teams. For this, leaders must be provided with sufficient resources to organise mobile work accordingly.

The managers themselves describe leadership from a distance as quite challenging. However, a range of opinions became clear here. In the group of managers, it was described that there is a great need for options for work design in order to organise mobile work in a healthy and productive way for the employees. The majority of managers emphasised that individual leadership behaviour is an important competence in the context of mobile work. It was therefore considered necessary that a future work agreement should not be over-regulated.

*“I know my staff well. I know who can easily cope with mobile work and who I have to support. I have to be able to decide individually in order to reach a good and fair agreement in the team about the design of mobile work in our section.”*

(Interview T1)

In connection with mobile work, difficulties were still reported in integrating new colleagues into the team. The reason given for this was the reduced personal contact.

*“I have only met my new colleagues virtually so far. Of course, it is difficult to really get to know someone”*

(Interview T1)

### 3.6. Advantages of Mobile Work

The most frequent mentions of advantages were the greatly reduced travel between home and work and the increased work-life balance. With regard to the journeys, not only the saving of travelling time was experienced as advantageous, but the stress caused by traffic jams, overcrowded roads, etc. was eliminated: *“I arrived at the company in the morning already exhausted before I had even started working”* (Interview T0). 

Mobile work would make *“regular sport possible”* (Interview T0) and the integration of *“ important private appointments (e.g., doctor’s appointment) uncomplicated”* (Interview T0). Other advantages were increased concentration and performance as well as higher motivation. An increase in quality of life and job satisfaction was expressed. Compared to working in the office, the working from home environment was quieter and less disruptive. As far as the external image of the company is concerned, the possibility of mobile work increases the attractiveness of the company for new skilled workers. From the perspective of the employees, mobile work sometimes makes it possible to avoid calling in sick when *“you’re not feeling well”* (Interview T0), because working from home is still possible *(“I can’t infect anyone with my cold”*) (Interview T0). Part-time workers reported that the elimination of travel times made it easier to reconcile family and work and made it conceivable to increase their working hours.

As already mentioned, the disadvantages mentioned were a reduction in social contact and a reduction in informal information that is important for work. The possibility that in the future, instead of individualised, quasi-personalised workplaces, a rotation procedure with shared desks could be introduced was predominantly connoted negatively.

The managers mentioned reduced physical contact as a disadvantage if employees were not feeling well. The problem of presenteeism was also critically discussed. Ach so

*“We simply need new ways of making contact with employees. I have to listen better and ensure regular conversations—I plan this much more consciously now than when I see people every day”*

(Interview T1)

### 3.7. Work Situation during the COVID-19 Pandemic

The greatly changed work situation during the COVID-19 pandemic was not representative of a regularly planned and implemented home-based work situation, came *“very suddenly”* (Interview T1) and was associated with a *“bumpy transition”* (Interview T1). With regard to the stresses and strains of the “ad hoc” changeover, the participants often reported initially experiencing a high level of stress. This is related in particular to technology-related frustration, deficits in work organisation and an inadequate working environment. However, this stress was almost completely eliminated in the course of time due to organisational and collegial support as well as an individual “habituation effect”. The majority of participants spoke of new and positive experiences and were positive about how well it had worked out in the end. The greatest stress in the context of the COVID-19 pandemic for participants remained a lack of childcare during the lockdown.

### 3.8. Implications for the Interfaces between Work Agreement and WHM 

Another important outcome of the study was to link the results between the work agreement and the WHM and to operationalise the important findings.

Within the framework of the study, as already explained, a qualitative evaluation and classification of the results was carried out together with the company—with a focus on the development of a work agreement and the implications for WHM. In the following, central conclusions are summarized in a table (Table 2).

## 4. Discussion

Finally, the results are discussed in light of the current literature and the strengths and limitations of the studies are presented. Furthermore, recommendations for practical implementation in companies and for future research will be derived.

The transformation of the world of work through digitalisation processes and new technologies is making work comprehensively more dynamic and leading to new or changed physical and psychosocial occupational risks [3,5]. These arise on the one hand from the dynamic interaction between interpersonal relationships (communication systems, social support, etc.) and on the other hand from the organisation of work (production rates, work schedules, workplace design) [4].

The results of our study correspond with these findings that mobile forms of work are particularly associated with changes at the level of work organisation as well as social interaction [1,11,46]. Demands arise at all levels of a company through flexibilised work processes up to the demands at the individual level of self-management [2,6,7,46]. 

In the literature, psychosocial risks of mobile work are discussed as an interrelation between risk exposure, social work context and work design [8,47] This assumption can be understood along the qualitative study data obtained here, as the need for action and the potential for design at all company levels becomes clear. 

Most of the findings of our study can be assigned to the level of work organisation and social interaction. This applies in particular to the organisation of working hours, cooperative collaboration with interdisciplinary work areas and in the team, aspects of accessibility as well as the provision of suitable work equipment in mobile work. This also corresponds with the results of other studies on mobile working [1,7,8]. Positive effects for employees include an improved work–life balance and increased freedom of action [7]. Negative experiences include reduced social interactions and trust deficits [46]. The focus group interviews allowed us to gain insight into the individual level of the employees in questions of self-organisation and self-management of their mobile work. This is closely related to the concept of job crafting, which is an important element of the Job Demands-Resources model (JD-R) and a theoretical basis of our study. Our qualitative results correspond with data from other studies that show that the freedom to shape one’s own work situation increases satisfaction and well-being [37,38]. However, in order to give employees this room for manoeuvre, transparent rules, agreements and trust are needed on the organisational side in addition to individual self-management competences. The reciprocal connection between personal competencies and organisational work design also became clear in the description of the experience of the work situation under pandemic conditions: the rather abrupt change to the WFH in the context of the pandemic-related lockdowns was primarily associated with uncertainty and stress, as it was described in the interviews. In the course of the pandemic, this experience of stress was almost completely reduced, as the employees received a lot of support, were in close contact with the leader and built up their own competences in dealing with the new situation.

Study results by Robelski et al. (2020) point to a deficit in terms of OHS structures and instruments in the field of mobile work, as OHS in companies is still very much oriented towards conventional work structures and physical presence of employees at the workplace [3].

In our study, employees were given the opportunity to participate in the design of criteria for a company agreement as well as indirectly for the derivation of occupational health management strategies. Through concrete feedback from the participants, criteria for both areas could be operationalised. From the authors’ point of view, the systematic approach of linking these two management systems presented in this paper has proven useful in generating important starting points for a health-oriented design of mobile work and serving as a starting point for further research. At the same time, the study wanted to make a contribution to the further development of WHM, as a lack of scientific knowledge is stated as to how WHM can be systematically integrated into the management levels of companies [26].

The results of the study give the participating company a comprehensive impression of the stresses, strains and resources of their employees due to mobile work, which can be addressed both by the work agreement and by WHM measures. 

For example, in the area of work organisation, the technical equipment for mobile work, times of availability or work processes in interdisciplinary cooperation can be regulated in principle and transparently within the framework of a work agreement. At the same time, further measures can be established from the various fields of action of the WHM, such as instruction in ergonomic work, management training or interdisciplinary workshops for the concrete coordination of cooperative collaboration. 

The results of this qualitative study are part of a longitudinal study in a mixed-methods design. The qualitative data gives us a deep insight into the complex organisational and social structures of the company as well as the corporate culture. This makes it possible to make very concrete recommendations for action for both the works agreement and the WHM, which could potentially significantly increase the fit of the measures through the procedure and especially through the participation of the employees. 

In Germany, work agreements are an important steering instrument for the design of mobile work. The results of our study could possibly be an impulse for companies to closely link work agreements with the WHM in the future. A systematic inclusion of the WHM at this management level could contribute to decisively increasing the health and safety of employees in mobile work. At the same time, this would be an opportunity for occupational health management to become more visible and anchored in important fields of action of a company.

## 5. Conclusions

Employee participation in strategic management processes through focus group interviews makes it possible to incorporate experiences, specifics, problems or even individual problem-solving strategies in a specific work context. Even though the subsequent evaluation of the implementation of the results of this study is still pending, data from other studies prove positive health effects of workplace prevention measures with direct or indirect participation of the employees themselves [8,48,49,50]. Early employee participation in change processes is also considered central to employee health and an important success factor for the success of change processes [51,52]. The present study shows that qualitative survey methods can be used in a resource-saving way and can contribute to the inclusion of health aspects in management processes, for example in the development of a work agreement. The results presented illustrate a close and synergistic interrelation between the design criteria of work agreements and employee health and occupational health management. They demonstrate how closely perceived aspects of organisational justice are associated with well-being and satisfaction: *“The criteria of who is allowed to do this and why, or not, must be quite transparent, otherwise stress arises”* (Interview T1). Well-being and satisfaction are important determinants of health. The intertwining of administrative and health-related issues suggests that approaches to healthy work design can or even must come from different areas in order to strengthen internal, health-related competences at all levels. Linking the approaches can create effective synergies and is a contribution to the development of a prevention and organizational culture.

## 6. Limitations

Like any study, ours had some limitations. In relation to mobile work, longer-term health effects cannot be assessed by the study design; longer periods of time would be necessary for this, which would allow the setting of a “steady state”. Qualitative studies could be assumed to have a lower degree of objectivity. To counter this, extensive quality assurance measures were applied in the survey and analysis procedure. The study presents implications and factors intrinsic to the organization for the healthy design of mobile work, the effectiveness of which has not (yet) been proven. This was not the aim of this study. Nevertheless, the evaluation of the effectiveness of the identified measures along the dynamic change process is desirable and will be pursued. 

## Figures and Tables

**Table 1:** A description of the samples (T0/T1).

	Survey T0	Survey T1
Time	09–10/2019	07–08/2020
Age (range)	21–61	21–65
Sex (in %) across all groups	46 male participants 31 female participants	59 male participants 45 female participants
Number of focus groups	13	16
Number of individual interviews	-	6
Group sizes min./max.	3–11 Participants	4–13 Participants
Total number of participants	77	104
Duration of group interviews	Ø 2–2 ½ h	Ø 2–2 ½ h
Duration of individual interviews	-	30–50 min

**Table 2:** Implications for work agreement and WHM based on the qualitative study results.

Main Codes	Implication for the Work Agreement	Implications for the Different, Interdisciplinary Fields of Action of WHM
work organization with provision of mobile and home-based work	➔ Determining the minimum equipment for home-based workplaces ➔ Ensuring timely online support (hotline)	WHM Field of action: Occupational safety and health protection of home-based workplaces ➔ Provision of suitable and healthy work equipment ➔ Instruction on ergonomic work
Documentation of working time/performance review	➔ Determine that performance and evaluation criteria should be provided by managers. ➔ Establishment of a contemporary time recording system ➔ Regular employee appraisals	WHM Field of action: Organizational and HR Development ➔ Agreements on accessibility ➔ Promoting a culture of trust through leadership development and appropriate options for transparent accessibility
Scope of application	➔ clear definition and setting of criteria for mobile work ➔ Job descriptions (inclusion and exclusion criteria) with decision-making leeway for managers	WHM Field of action: Organizational and HR Development ➔ Capacity building of leaders on distributive justice and participation. ➔ Provision of rooms at the office for meetings
Self-organization/individual work design ability	➔ Regular employee appraisals	WHM Field of action: Human resources development ➔ Increasing & strengthening personal competencies and work design skills ➔ Social counselling (EAP)
Contact with the workplace and social interaction incl. leadership	➔ Establishment of transparent information and communication structures ➔ Regulations on interdisciplinary interfaces	WHM Field of action: Organizational and trust culture ➔ Competence development for managers, especially on virtual leadership ➔ Interdisciplinary workshops for cooperation
Health related benefits	➔ Appropriate scheduling of working from home (e.g., regulations based on whole days to reduce car journeys)	WHM Field of action: Workplace health promotion ➔ Promotion of work-life balance ➔ Strengthening personal responsibility in dealing with the phenomenon of presenteeism at working from home
COVID-19-related influences	➔ Special arrangements for pandemic	WHM Field of action: Human Resources Development/Organizational Development ➔ Promotion of organisational change competencies ➔ Promotion of individual change competencies

## Data Availability

The data that support the findings of this study can be requested from the corresponding author, [M.J.], upon reasonable request.
